# Craniometric Characteristics of Selected Carnivora Species Kept in Captivity in Relation to Bite Force and Bending Strength of the Upper Canines

**DOI:** 10.3390/ani14091367

**Published:** 2024-05-01

**Authors:** Katarzyna Różycka, Ewa Skibniewska, Łukasz Rajkowski, Michał Skibniewski

**Affiliations:** 1 Department of Morphological Sciences, Warsaw University of Life Sciences, Nowoursynowska 159C, 02-776 Warsaw, Poland; michal_skibniewski@sggw.edu.pl; 2 Department of Biology of Animal Environment, Warsaw University of Life Sciences, Ciszewskiego 8, 02-787 Warsaw, Poland; ewa_skibniewska@sggw.edu.pl; 3 Mathematical Statistics at the Faculty of Mathematics, Informatics and Mechanics, University of Warsaw, ul. Banacha 2, 02-097 Warsaw, Poland; l.rajkowski@mimuw.edu.pl

**Keywords:** bite force, Carnivora, skull, temporomandibular joint

## Abstract

**Simple Summary:**

This study aimed to analyse morphological parameters related to the bite apparatus in representatives of seven families of captive animals. Thirty-three measurements were taken of the skull, mandible, and surfaces of the masseter and temporalis muscles. Some were used to calculate the bite force on the maxillary canine and molar teeth. A high correlation between bite force and animal size was confirmed. The bending strength of the upper canines was determined using their lateromedial and anteroposterior dimensions.

**Abstract:**

The aim of this study was to analyse the bite forces of seven species from three carnivore families: Canidae, Felidae, and Ursidae. The material consisted of complete, dry crania and mandibles. A total of 33 measurements were taken on each skull, mandible, temporomandibular joint, and teeth. The area of the temporalis and masseter muscles was calculated, as was the length of the arms of the forces acting on them. Based on the results, the bite force was calculated using a mathematical lever model. This study compared the estimated areas of the masticatory muscles and the bending strength of the upper canines among seven species. A strong correlation was found between cranial size and bite force. The results confirmed the hypothesis that the weight of the animal and the size of the skull have a significant effect on the bite force.

## 1. Introduction

The dentition of most representatives of the Canidae and Felidae family members is marked by reduced incisors and defined powerful canines. Molar teeth are adapted predominantly to biting or grinding (in the case of brown bears) food. Morphological classification defines cheek teeth as secodont-type dentition. Unlike other mammalian species, carnivores do not need to thoroughly grind their food to consume it [1,2]. Canine teeth play an important role in enabling the grasping of prey and defence against danger. For this reason, these teeth are often damaged during fighting or hunting behaviour [3]. A pivotal role is also played by a carnivore-specific pair of teeth called the carnassial, designed to shear off pieces of food. The Carnivora order encompasses two suborders: Caniforma, which includes, among other things, canids and bears, and Feliforma, which includes, among other things, cats and hyenas [4].

### Masticatory System in Canidae, Felidae, and Ursidae

All components of the masticatory apparatus act as knives to cut food. The lower jaw acts as a movable bar and unilateral lever that applies forces to the dentition. These relationships allow a mathematical analysis of the function of the occlusal apparatus [5]. The ability to consume food depends on the proper functioning of the relevant structures. Pathologies associated with gum and periodontal disease can cause pain and weaken bite force. Damage and inflammation limiting the mobility of the mandible, as well as deficiencies in the dentition, are key determinants for the survival of animals living in the wild. The morphology, mechanics, and forces operating throughout the temporomandibular joint have been described in several publications over the last few years [6,7,8,9,10,11,12,13]. Because of the data published, it seems that new facts on its functioning mechanism are still being discovered, despite its apparent anatomical simplicity. Estimating the forces generated in the masticatory system is a particularly important issue. Despite the available in vivo measurement methods, it should be noted that they are burdened with inaccuracies related to the impact of the specimen’s consciousness. Much more reliable data can be obtained by conducting an analysis of the morphology and biomechanics of the joint structures, dental arches, and the muscle system involved in their movement. So far, bite force has been analysed based on measurements of the area of masticatory muscles, such as the masseter and temporal muscles. Using the beam and lever arm model, Thomason [5] designed a method to estimate the mammalian bite force. It allows for the analysis of macerated bone material; hence, it enables the analysis of the above-mentioned factors in extinct species. Published research on the mathematical modelling of forces in the masticatory system has led to an increased interest in the subject.

The second factor that should be taken into consideration is the bending strength of canines, since in carnivores, resistance to external forces determines their ability to survive. Injury leading to fracture of canine crowns limits hunting effectiveness and the ability to defend an individual itself and the defence of its territory as well.

An important issue is also the possibility of making interspecies comparisons of the results obtained using objective criteria without reference to absolute values, which vary significantly due to the diversity of body sizes of the individuals studied.

The aim of this study is to compare the ecomorphology of particular carnivoran species based on two proxies: bite force and upper canine bending strength. These two proxies are very central in the ecology of the carnivorans since they can be associated with diet and defence, and they can affect their different ecological roles. The additional aim of this study was to compare results obtained from members of different carnivore species kept in captivity with those obtained from free-living individuals. Of particular interest are the results for species kept in zoos, as hybridisation was a common breeding practice many decades ago. Therefore, it is worthwhile to compare the results of these individuals to determine whether breeding processes have affected the masticatory apparatus in terms of the generated bite forces and the bending strength of the dentition.

## 2. Materials and Methods

The research material consisted of skulls kept in the museum resources of the Department of Morphological Sciences of the Institute of Veterinary Medicine at Warsaw University of Life Sciences. Five skulls per representative of the mesocephalic Canidae (domestic dog—*Canis lupus familiaris*, red fox—*Vulpes vulpes*, grey wolf—*Canis lupus*), Ursidae (brown bear—*Ursus arctos*), and Felidae (domestic cat—*Felis catus*, African lion—*Panthera leo*, and three skulls of the Bengal tiger—*Panthera tigris tigris*) families were used in the study. The skulls were taken from individuals kept in captivity. Specimens were selected according to the cranium–mandible and dentition completeness. Therefore, they appear to be optimal research tools in relation to macerated bone preparations maintained in the collections of museum research centres.

The cranial structures were examined with both traditional measuring devices and a digital system. All structures were captured with a Nikon D3400 camera (Nikon, Tokyo, Japan). The structures used as landmarks were selected based on studies by von den Driesch [14] and Christiansen and Adolfsen [15].

A total of 33 measurements of each cranium, mandible, temporomandibular joint, and teeth were taken using a digital caliper. All procedures were documented with photographs. Each measurement was taken three times by the same person, and then the average obtained from the measurements was taken as the value for further data analysis. Measurements were also taken with the use of the Axiovision™ system, in which images were recorded in the presence of standardised distance markers with an accuracy of 0.01 mm.

The cranium measurements included the distance between the alveolar process at the first incisor to the occipital condyles (UDAL), neurocranium length (UDAC), viscerocranium length (UDAF), the widest distance between mastoid processes of the temporal bone (UDAMM), the widest palatal dimension measured at the alveolar processes of the P4 (UDAP4), and the dimension measured at the canines (UDACa). The cranial height (UDAH) in felids corresponded to the occipital squama height in canids and Ursidae.

The mandible measurements included the length of the mandible (LDA L) and the distance between the condyloid process and the mandibular angular process (LDAPP).

The height and width of the mandible and maxilla articular surfaces and temporomandibular joint were measured (UDA/LDA TMJ H/L).

**Figure 1:**
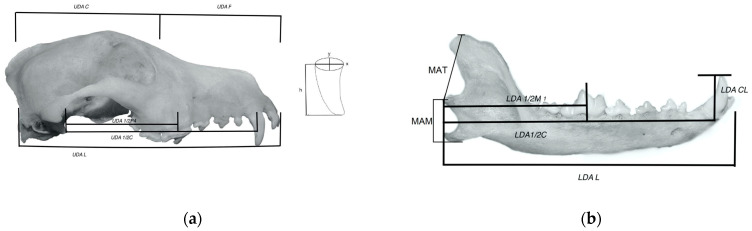
(**a**) Skull of *Canis lupus familiaris*, illustrating measurements. (Upper dental arch: length of the skull—UDA L, cranium length—UDA C, face length—UDA F, length from the joint process to half P4—UDA 1/2P4, length from the joint process to half canine—UDA 1/2C, length of the crown—h); x—aneposterior radius of the canine, y—lateromedial radius of the canine, both measured at the gum line. (**b**) Mandible of *Canis lupus familiaris*, illustrating measurements. (Lower dental arch: length of the jaw—LDA L; length from the joint process to half M1—LDA 1/2M1; length from the joint process to half canine—LDA 1/2C; length of the crown—LDA CL; MAT—moment arm of the temporalis muscle; MAM—moment arm of the masseter muscle).

The dentition parameters were the length from the articular process to mid-maxilla P4 (UDA1/2P4) and mid-mandible M1 (LDA1/2M1), as well as the length to the middle of the upper and lower arch (UDA/LDA 1/2C). Crowns of the canines (LDA/UDA CL), their aneposterior (x), and lateromedial radius (y) at the gum line were also measured. (Figure 1). All of these measurements were taken on either side (right or left) [15].

The museum did not provide information on the live weight of the specimens studied. To ensure accuracy, we used Engelman’s [16] method of calculating body weight (BW) from the distance between the bilateral occipital condyles (OCW). The results obtained in other publications [17,18] were analysed, and then an individual point distribution fit was performed based on the OCW-based weight estimation.

**Figure 2:**
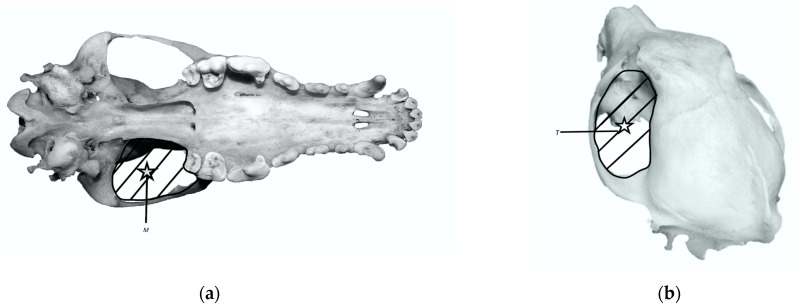
(**a**) Reconstruction of cross-sectional areas in the masseter–pterygoid group (M); (**b**) reconstruction of cross-sectional areas in the temporalis muscle (T).

The bite force was measured using the lever model developed by Thomason et al. [1]. Images of skulls in the caudal-dorsal position were prepared in Axiovision™ 4.8 software. The surface of the masticatory muscles, temporalis, and masseter, as well as the length of the arms of forces acting on them, were calculated. The surfaces of the masseter (MT) and temporalis (TT) are expressed in square centimetres (Figure 2). The length of the masseter (MLine) and temporalis (TLine) lever arms was measured from the geometric centre of the respective muscle to the articular surface of the temporomandibular joint (TMJ). They are expressed in centimeters. Depending on the landmark, the distances between TMJ and the canine (OC) or the first molar (OM1) were also determined.

The total bite force was calculated using the following formula [15]:

CBF = (2·(MT·ML + TT·TL)·FPA)/Oc(1)

MBF = (2·(MT·ML + TT·TL)·FPA)/Om(2)

Force values were expressed in Newton units (N). FPA stands for force per cross-sectional area of muscle. It is considered constant in mammals (300 KPa per 1 cm^2^) [15].

Bite force for canines (CBF) and carnassial specific to the species (MBF) were measured on both sides, right and left.

The relationship between bite force and body mass in the studied carnivoran families is allometric. That is why bite force values do not increase with body mass. For this reason, the bite force quotient (BFQ) was used to compare bite forces between different animal species. This value allows comparison of bite forces in species with different body sizes. In the present study, the Christiansen, Wroe, and Hite methodology [16,17] was used to calculate the BFQ. The bite force quotient was calculated from equations of the residuals of a regression analysis of the estimated bite forces at the tip of the canine and middle of the breaker tooth, respectively, to the estimated body weights. The following formula was used:

CBFQ = 100 × CBF/10^(0.583·log(BM)+0.1458)^ (3)

MBFQ = 100 × MBF/10^(0.575·log(BM)+0.1468)^ (4)

where CBF and MBF are in Newtons and BM is in grammes.

Data on bite force and body weight in the studied animals were logarithmically transformed by the model proposed by Van Valkenburgh and Ruff [19] and Christiansen and Adolfssen [15].

The second moment of area for bending was calculated using the following formula [15]:

I_AP_ = (π·x·y^3^)/4 (5)

and I_LM_ = (π·y·x^3^)/4 (6)

where AP and LM are the anteroposterior and lateromedial axes of the upper canine. x and y are, accordingly, the anteroposterior and lateromedial radii of the canine. They were measured at the junction of dentine and enamel. Peak bending strength (S) was calculated as:

S_AP_ = I_AP_/F·h·y (7)

and S_AM_ = I_LM_/F·h·x (8)

where h is the crown height, the F value is set at one, and it has no effect on the result.

The statistical analysis of the results obtained was developed using Statistica 13.3 software (TIBCO Inc.™, Palo Alto, CA, USA).

## 3. Results

The analysis confirmed the claim made by Ellis and Thomason [20] that there is a close correlation between the size of an animal and the shape of its skull. The museum records did not include information on the weight of the animals studied. Therefore, the body masses were estimated using the OCW and Engelman’s methodology [16] (Table 1). Sexual dimorphism was not taken into account in these values.

In the two large felids included in this study, the lion and the tiger, the average length of the skull was 300.12 mm and 293.350 mm, respectively. The respected value for domestic cats was 88.762 mm. In canid representatives of the wolf, fox, and domestic dog, they were, respectively, 245.082 mm, 138.812 mm, and 176.642 mm. In the analysed bears, the average cranial length was 316.112 mm. The length of the mandible also increases in proportion to the dimensions of the cranial length and is 223.888 mm in the lion, 213.930 mm in the tiger, and 58.222 mm in the domestic cat, respectively. This fact is also unchanged in the wolf (179.812 mm), fox (105.446 mm), domestic dog (133.722 mm), and bear (220.69 mm).

A comparison of measurements of the articular surfaces of the temporomandibular joint showed that the length of the condylar process corresponded to the length of the articular surfaces on the temporal bone in the cat, tiger, and fox. This difference in the lion, dog, wolf, and bear was about 1.153 mm, 2.399 mm, 3.961 mm, and 2.114 mm, respectively. There are no significant differences between the species studied, probably as a result of the lack of lateral movement of the mandible in carnivorans. Significantly greater differences were observed in the width of the contact surface of the condylar process in lion, bear, tiger, dog, wolf, and fox: 6.38 mm, 5.92 mm, 5.91 mm, 5.08 mm, 4.98 mm, and 3.05 mm, respectively. The smallest difference in these dimensions occurred in the domestic cat and amounted to 1.73 mm. This stems from the fact that this space, equivalent to the articular cavity, is filled by an articular disc that facilitates the opening and closing of the oral cavity. Analysing the above dimensions, it can be concluded that the articular disc of the temporomandibular joint is a subject of significant deformation along its surface.

Force measurements were taken on either side at the canines (C) and first molar (M1) (Table 2).

The average values of canine bite force in the groups studied were as follows: Among the cat family, tigers (3375.27 N) and lions (2608.77 N), domestic cats have a lower bite force of 184.06 N. In the canid family, the average values for canine bite force were 1141.26 N for the grey wolf, 540.56 N for the domestic dog, and 344.35 N for the red fox. The value of canine bite strength for the brown bear ranked between the largest felines and canids and reached 1627.01 N.

The average bite forces on carnassials in the groups studied were as follows: Among the cat family, tigers (4172.26 N) and lions (4566.87 N) have the strongest bite forces. Domestic cats have a lower bite force of 332.79 N. In the canid family, the average values for bite force on M1 were 2425.8 N for the grey wolf, 1100.59 N for the domestic dog, and 774.15 N for the red fox. The value of canine bite strength for the brown bear was 3175.99 N. In all studied individuals, the bite force on the carnassial is, on average, 1.7–2.2 times greater than the bite force generated on the canine. 

This observation applies to all the specimens studied. Analysing the anatomical structure of the masticatory muscles, we find that the temporalis muscle has a larger surface area than the masseter muscle. Analysing the results of the measurements, we can see that the measured areas differ, and the masseter muscle is the larger one. This may have to do with the fact that the surface area of these muscles is measured theoretically at the places of their greatest thickness. The bite force formula given by Thomason is used to analyse dry skulls when we do not have access to wet specimens. By measuring the bones of the skull, we can estimate the span of the masticatory muscles, but we cannot calculate them accurately (Table 3).

However, these are average weights and do not take overweight or underweight into account. Considering the absolute force acting on the canines and molars in relation to the absolute weight of the animal, an interesting conclusion can be reached. Namely, it turns out that the domestic cat has the highest absolute force acting on both canines and molars of all the felids studied, while in canids, the red fox has the highest absolute force in relation to body weight. The absolute bite force in the species studied decreases as the animal grows. This results in a kind of erroneous assumption. This is because it is impossible to compare individuals with diametrically opposed body weights, which is why the BFQ was used. As explained in Wroe et al. [21], Christiansen [15], Hartstone-Rose et al. [22], and Damasceno et al. [23], the absolute values of bite force need to be adjusted in order to enable interspecific comparisons. Analysing the selected values in relation to body weight, it can be concluded that by far the highest bite force is possessed by the domestic cat, which has 40.9 N of bite force per 1 kg of body weight, while in the lion, this value is only 16.72 N. Bite force changes in an allometric manner, adapted to the hunting behaviour of a given individual in the context of the size of potential prey and the force required to obtain it. Assuming a simple translation of the values recorded in the cat to the body weight in the lion, such an individual should bite with a force of 6380.4 N, which would certainly have a significant impact on the dentition and cranial skeleton, which would have to be characterised by a morphology that would make it possible to compensate for such enormous forces generated in the bite apparatus.

Analysis of the bite force quotients on canines, relative to body weight, entitles us to conclude that among the studied felines, the Bengal tiger bites the strongest (193.46), followed by the African lion (119.46) and domestic cats (79.19). Analysing MBFQ, the Bengal tiger has the highest bite force on breakers (132.59), the African lion is next (117.25), and the domestic cat (69.69) is the last one. In canids, the highest CBFQ is the red fox (141.29), the next grey wolf (100.60), and the domestic dog (90.42). MBFQ increases in the same order (155.17, 115.38, 94.17). CBFQ and MBFQ for brown bears are 76.07 and 83.56, respectively. Outliers were ignored when calculating the average BFQ.

The average bending strength, in AP directions, is highest in the lion, followed in descending order by the values for bear, tiger, wolf, dog, fox, and cat. The average bending strength, in LM directions, is highest in the bear, followed in descending order by the values for the lion, tiger, wolf, dog, fox, and cat (Table 4).

**Figure 3:**
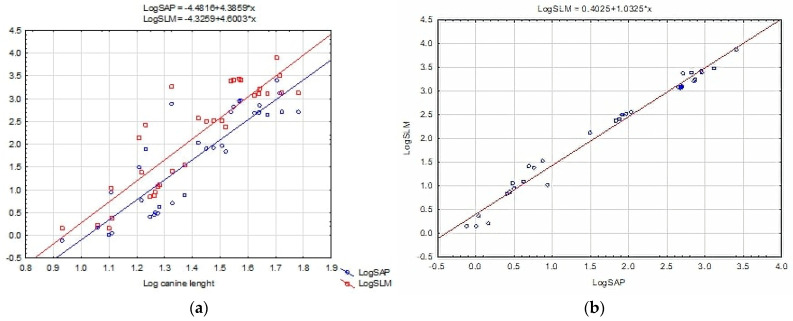
Log–log plot of (**a**) bending strength in AP and LM directions against canine length; (**b**) lateromedial bending strength to anteroposterior bending strength.

For clarity in illustrating the results, the data in the plots were made into logarithms. Bending strength in the AP and LM directions increases as the canine crown lengthens in the specimens studied in Figure 3a. As bending strength increases in the AP direction, the corresponding value in the LM direction increases (Figure 3b).

**Figure 4:**
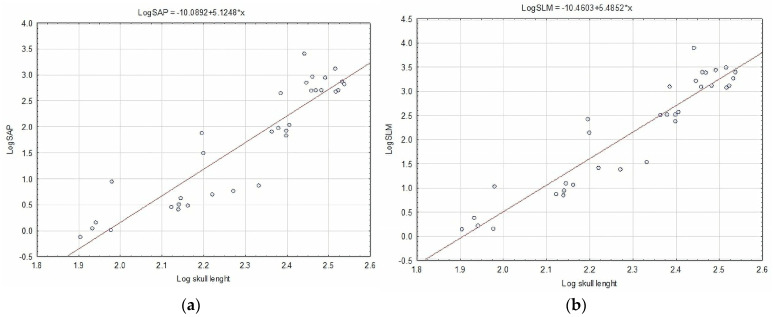
Log–log plot of (**a**) bending strength in AP direction; (**b**) LM direction to skull length.

As the cranial length of the studied specimens increases, the bending strength in the AP and LM positions increases (Figure 4).

The surface areas of the masseter and temporalis muscles were measured at the point of their greatest thickness, so the weights of the masseter muscle are, on average, 0.8–1.3 greater than those of the temporalis muscle (see Figure 2). The values for MT and TT in each species, arranged in descending order, are as follows: African lion (97.12 cm^2^, 78.80 cm^2^), tiger (75.93 cm^2^, 80.31 cm^2^), brown bear (56.95 cm^2^, 49.53 cm^2^), grey wolf (34.60 cm^2^, 35.90 cm^2^), domestic dog (20.24 cm^2^, 15.29 cm^2^), red fox (10.49 cm^2^, 11.90 cm^2^), domestic cat (8.00 cm^2^, 6.42 cm^2^).

## 4. Discussion

The temporomandibular joint and the morphology of carnivore dentition have been the topic of numerous studies in recent years [6,7,8,9,10,11,12,13,15]. A large number of studies dealing with topics from the borders of comparative anatomy, dentistry, and the mechanics of the masticatory system were published. The particularly important role of the research comes from the possibility of analysing the function of the bite apparatus in relation to the hunting behaviour of prey animals in the context of prey size and hunting specialisation. Thomason’s work represents a significant contribution to this field [1]. In his paper on opossums, he suggested an innovative approach to the analysis of the masticatory system and the forces generated by its elements. The authors of this paper are aware that the proposed model has certain limitations, but by using bone material, it is possible to objectify the results. As mentioned above, in vivo studies are also subject to inaccuracies due to the influence of the subject’s consciousness or current state of health. The presence of inflammation, erosions on the gums, and cavities in the enamel can result in pain and discomfort when eating. These seemingly minor ailments can have a significant impact on the animal’s bite force and its ability to obtain food. It is well known that not every bite is delivered with equal force. It is also known that an animal with, for example, a chronic metabolic disease that causes temporal muscle atrophy will produce less force. This is why this method was chosen. Building on the model proposed by Thomason [1], Christiansen and Wroe [17] proved that the bite force of an individual depends, among other things, on its body mass. They proved that, in relation to BM, smaller species had stronger jaws than larger species. The examples of the giant panda (*Ailurupoda melanoleuca*) and the red panda (*Ailurus fulgens*) also demonstrated the correlation between diet and bite force. By adapting to consuming bamboo, these animals developed a masticatory system that generated forces much stronger in relation to their body mass than other species of a similar size. These results are in line with the data collected in the author’s own studies. An exception to Christiansen’s and Wroe’s theory (other than the pandas they specified) is the results for bears concerning cranial length and bite force in relation to mass. The bite force in the Ursidae family is relatively low compared to its representative, BM. The bite force of specimens weighing, on average, 140kg ranges from 0.8 to 2.6 thousand N at the canines and 1.9 to 5 thousand N at the carnassial. These findings contrast with the results obtained in large felids, namely lions and tigers. In these animals, with an average weight of approximately 145 kg and 99kg, the bite force at the canines is roughly 1.7–2.7 thousand N for lions and 3.2 thousand N for tigers. At carnassial bite force values of 3.2–3.9 thousand N for lions and 2.9–3.6 thousand N for tigers. It is nearly twice as high in relation to their body mass as in the case of bears. This is due to a different food base. However, felines are obligatory predators. The brown bear’s diet includes both plant and animal foods. Plant food is represented by the fruits of forest shrubs, which does not require an evolutionary investment in strong dentition [19]. In the other analysed species, the estimated bite force is directly related to the animal’s size and diet. The bite force of smaller cats, such as domestic cats, is around 219–126 N on canines and 407–217 N on carnassials. This is directly related to the reduced size of the animal and its skull. In canids, the canine bite force of wolves and dogs ranges from 423 to 783 N and 846 to 1.2 thousand N on M1, 870 to 1.2 thousand N, and 910 to 1.4 thousand N, respectively; in foxes, it is merely 331–224 N and 516–1.3 thousand N. Ellis et al. [20] analysed bite force in relation to the size and type of skull of a domestic dog. It was noted that the length of the lever used to measure BF varied depending on the morphological type: mesocephalic, brachycephalic, or dolichocephalic. It was confirmed that bite force rises with the size and length of the skull, and as predicted, its value was higher at M1/P4 than at the canines. Significant deviations in this field were observed in the bears analysed. Their bite force is low in relation to skull length. This phenomenon may be explained by the fact that the masticatory muscle surface of the species is generally small in relation to the skull size.

The number and shape of teeth vary not only between but also within the different families of carnivorous mammals. The type of food consumed, individual size, and position in the food chain influence the functionality of the masticatory system. Canids and bears have 42 permanent teeth, while felids have 30 [8,24]. 

The skulls examined had no missing teeth, for example, due to the absence of a permanent tooth bud. An anomaly observed in over half of the examined specimens was attrition of enamel resulting from natural causes. Abrasion due to mechanical damage, e.g., crown fracture, was less common. In felids, attrition was observed in eight out of sixteen studied skulls, while abrasion was detected in seven. In canids, attrition was visible in seven specimens, while abrasion was observed in only one of them. This group consisted of fifteen specimens in total. The five specimens of Ursidae had mostly worn enamel (four) and broken crowns (two). Based on the analysis, it can be concluded that canines and molars are the most vulnerable to surface lesions and abrasion. The canines of the upper dental arch were, on average, 5–20% longer than their lower arch counterparts. This is due not only to the maxilla–mandible size ratio but also to the fact that they serve different purposes. Canines are designed not only to capture and hold prey but also to deter any potential rivals [17].

The temporomandibular joint is pivotal for the proper functioning of the masticatory system. Its mechanics rely on the close cooperation of the largest masticatory muscles: the masseter, the pterygoid muscles, the temporalis, and the digastric muscle. Any lesions in this area may be painful, impair food intake, and consequently lead to the death of an animal living in the wild. Research on this subject was conducted by Arzi et al. [25]. They found that temporomandibular joint problems are very common among dogs. It is worth noting that out of 15 patients exhibiting only degenerative lesions in CT, medical symptoms occurred in only 4. These findings suggest that temporomandibular joint disorders are not diagnosed in many dogs. The discomfort and pain accompanying such lesions are often deadened by the survival instinct. Nevertheless, in the present study, the authors have not observed any symptoms of pathological changes in the skulls studied. This is in contrast to a study by Arzi et al. [25]. It can be concluded that the pathologies of the TMJ are more associated with soft tissue than with the osseous structures forming this joint.

The next part of the masticatory system is the masseter muscle, which is responsible for elevating the mandible. In carnivorans, this muscle is thick and convex, covering the external surface of the mandible ramus. The temporal muscle is the largest muscle of the head in carnivores. It is very long and broad and fills the entire area of the temporal fossa [26].

Lateral and medial pterygoid muscles run from the sphenoid bone, pterygoid hamulus, and pterygoid bone, inserting into the medial surface of the ramus. Together with the temporal muscles, they enable mandibular elevation and protrusion [27]. In analysed research studies, pterygoid muscles are usually only mentioned. None of Thomason’s mathematical analyses consider their operations as separate units. They tend to be measured as a part of the masseter muscle. This could be due to the fact that these muscles are small and narrow compared to the superior size and volume of the masseter and temporalis. Their location under the other muscles and difficulties in determining their position on a dried, medically prepared skull may also have an impact.

Macerated bone material enabling the analysis of masseter and temporalis surfaces was used for analyses performed during the author’s own studies. The muscles showed two-sided symmetry and comparable surfaces in all cases studied.

This study shows that in all examined specimens of the Carnivora order, the ratio between the surface of the masseter and the surface of the temporal muscle is 50–33% smaller on average for all examined specimens. A larger surface and muscle mass of the temporal muscle lead to its greater strength. The author’s own studies do not support this thesis. Analysis of data showed that in tigers, the masseter muscle surface was larger (by 5.45%, on average) or equal to that of the temporalis. Similar results were found in foxes (11.84%) and wolves (5.01%). The values observed may be linked to a larger length–height ratio of the skull. This observation is worth noting and analysing in future studies since a large skull and body size do not necessarily implicate a large volume of masticatory muscles, as was found when examining the skulls of bears. Similar conclusions about the size of masticatory muscles were reached by van der Meiden et al. [28] in a study of bite forces in lions and tigers using an in vivo method. Their results indicated that bite forces were higher than those predicted by the dry skull measurement method [3,16,23]. It was concluded that this underestimation of results was due to a flaw in the dry skull measurement method. It is an error that overestimates the physiological cross-sectional area of the masseter muscle and underestimates the physiological area of the temporalis muscle. It is also recommended to standardise the methodology of measurements of the masticatory muscles used to calculate BF. In the measurements used to determine BF, the areas of the masticatory muscles were measured at the point of their greatest thickness, stimulating the extent of these areas (comparing diagrams and photos) in different authors [1,15,17,19]. Therefore, it is possible that there are differences in the average dimensions of the areas of individual muscles.

The use of Engelman’s [16] methodology to determine body mass is very helpful, especially in extinct species in which we have no knowledge of how much their body mass was. When comparing data from the literature and the average weights of the species we studied (using the formulas for carnivores from Engelman’s [16] publication), it can be seen that some species may have under/overestimated body mass values. This is the case of the grey wolf, whose BM values calculated on the basis of the OCW and compared with the data of the aforementioned author are significantly higher than the averages reported in the literature. Therefore, in order to be as accurate as possible in calculating the mass of a given species, it is worth analysing them individually but still with reference to all predators, i.e., using the graph given by Engelman [16]. Therefore, a new prediction curve has been created in our own compilation of the results, adapted to the species in relation to the Engelman [16] curve and his OCW and BM values. The new curve was created using a logarithmic model. During the analysis, a problem was experienced in estimating the mass of the African lion, as Engelman [16] only calculated values for one individual, while Bengal tigers, cats, and domestic dogs were not included at all. In order to predict the masses of the big cats, data on the Pantheridae were used, and a curve was calculated, which was used to calculate the BM in African lions and Bengal tigers. To calculate the presumed body masses of the domestic dog, grey wolf data were used due to the fact that the domestic dog is a form of grey wolf.

The bite force quotient is a value calculated as a regression of the quotient of an animal’s bite force divided by its body mass. The BFQ was first used by Wroe et al. in 2005 in an article [21] on extinct and living carnivorous mammals. That work compared the bite strengths, body weights, and prey sizes of carnivores. The study was expanded two years later in a paper by Christiansen and Wroe [17]. The results of both works showed that predators that take relatively large prey have high bite forces relative to their size, a finding that was also confirmed during our study. The bite force is also influenced by the type of food taken and its size. Omnivores feeding on small prey are similar but well below the value of carnivores feeding on large prey. Large prey relative to the body weight of the carnivore requires a large BF, as confirmed in our studies in the Felidae family. BFQ unifies the data obtained and allows comparison of individuals with different body sizes, among others. Hite et al. [18] used the BFQ to compare bite forces measured in vivo in mole rats of different castes with average bite force values of 82 mammalian species. The author noted the effect of awareness, duration measurements, and test conditions on live animals. His results were balanced with the predicted values from cranial measurements, which so far came out much higher than in vivo. The authors’ study found that the estimated BFQ values were lower than those reported in the literature. This suggests that captive animals may have a lower bite force than their free-living counterparts. The reason for this could be that captive animals do not have to hunt and have constant access to food, unlike wild animals, who experience periods of starvation. This difference has a significant impact on the parameters recorded in the bite apparatus.

**Figure 5:**
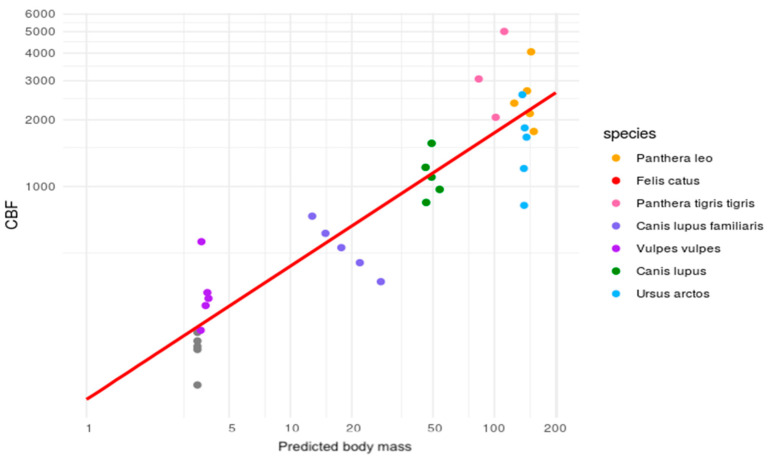
Bite force quotient estimations for seven species.

The authors’ study also found a surprisingly high BFQ for the red fox, which differs from other publications. This difference may be due to the fact that the BFQ depends on the entire group on which the regression was performed. It should be noted that many species may change the relationships between specific groups compared to the same analysis of a smaller number of species. This observation is supported by Figure 5.

The method of calculating the BFQ is one of wonder. Hite [18], citing an article by Christiansen and Wroe [17], used different units when sub-estimating the mass of specimens studied, namely grammes instead of kilogrammes. The paper below also encountered a problem with standardising units, and the use of grammes allowed for standardisation and increased the similarity of the results obtained with earlier literature. This is an issue worth exploring in future analyses.

Analysing bending strength (SAP/SLM) in the studied species confirmed the statement of von Valkenbourgh [19] that SLM is greater than SAP. The smallest differences between the above values are found in small dogs and cats. Despite this, cats have stronger canines in relation to their crown length than dogs. Comparing SLM, if a dog had its value similar to a cat, its canine should be about 37.8 cm long, while in reality, dogs have canines with an average length of 1.8cm and cats 1.67 cm. Certainly, these differences are influenced by the shape and size of the canines, as well as the hunting method and the size of the prey in relation to the body mass of the predator. Despite this, crown fractures in cats are relatively common, especially in males. During hunting, cats catch their prey in the laryngeal area and, with strong strokes of the neck, strangle them while breaking the victim’s neck [29].

## 5. Conclusions

In conclusion, when analysing bite force generated at the canines and carnassial, similar values were found to those reported by Thomason et al. [1,5,20,24]. It is striking, however, that none of the cited researchers examined the forces acting directly on the surface of the temporomandibular joint. This is a topic that should be addressed in the future, as these forces affect the wear of the articular disc and the correct functioning of the joint as a whole. This study showed differences in the morphology of dentition and of the temporomandibular joint between the examined families of Carnivora. It is particularly worth noting that, according to the studies, the temporalis muscle is not always the dominant masticatory muscle. The differences arise not only from the different body structures, skulls, and sizes of the examined specimens but also from adaptations to the type of food consumed. Individuals in captivity were found to have a lower BFQ than their wild counterparts, as reported in the literature. It was observed that the forces generated at the breakers are almost twice as high as at the canines. This is directly related to the increased frequency of attrition of molar crowns. This phenomenon explains fractures of molar teeth and periapical abscesses, which can be observed in carnivorous animals. This increased load and almost double the forces acting on the structures of the periodontium may weaken them and directly contribute to the formation of pathology. These findings confirm the need for further research to better understand the mechanics of masticatory muscles, the temporomandibular joint, and the forces acting within it. The literature shows a high prevalence of various lesions in the temporomandibular joint [10,11,12]. Therefore, studies on the links between the morphology and mechanics of the various parts of the masticatory system have a useful value, allowing clinicians to broaden the knowledge necessary to diagnose and treat masticatory system disorders.

## Figures and Tables

**Table 1:** Width values between the occipital condyles (OCW) in mm and the corresponding body mass (BM) in g.

Order	Taxa	OCW	BM
**Felidae**	***Panthera leo***	60.42	125,140
	***Panthera leo***	65.54	155,992
	***Panthera leo***	64.81	151,329
	***Panthera leo***	64.47	149,188
	***Panthera leo***	63.73	144,594
**Felidae**	***Felis catus***	22.62	3155
	***Felis catus***	22.72	3210
	***Felis catus***	21.57	2618
	***Felis catus***	21.5	2585
	***Felis catus***	21.46	2566
**Felidae**	***Panthera tigris tigris***	57.95	111,760
	***Panthera tigris tigris***	55.92	101,468
	***Panthera tigris tigris***	52.11	83,811
**Canidae**	***Canis lupus familiaris***	35.53	14,847
	***Canis lupus familiaris***	33.95	12,784
	***Canis lupus familiaris***	37.52	17,763
	***Canis lupus familiaris***	42.97	27,752
	***Canis lupus familiaris***	39.98	21,890
**Canidae**	***Vulpes vulpes***	22.34	3632
	***Vulpes vulpes***	23.86	3839
	***Vulpes vulpes***	24.43	3915
	***Vulpes vulpes***	22.55	3661
	***Vulpes vulpes***	24.79	3964
**Canidae**	***Canis lupus***	50.1	45,988
	***Canis lupus***	51.13	49,172
	***Canis lupus***	51.15	49,236
	***Canis lupus***	52.58	53,911
	***Canis lupus***	50.2	46,291
**Ursidae**	***Ursus arctos***	62.55	139,525
	***Ursus arctos***	63.29	143,716
	***Ursus arctos***	62.61	139,862
	***Ursus arctos***	62.77	140,764
	***Ursus arctos***	62.12	137,125

**Table 2:** Median values of basic craniofacial and mandibular measurements (mm).

Order	Taxa	UDA Length	UDA Width	LDA Length	UDA Canine Length	LDA Canine Length
**Felidae**	***Panthera leo***	300.12	258.08	223.89	46.48	40.26
**Felidae**	***Felis catus***	88.76	76.24	58.22	11.68	10.07
**Felidae**	***Panthera tigris tigris***	293.35	245.40	213.93	53.60	46.38
**Canidae**	***Canis lupus familiaris***	176.64	114.10	133.72	18.95	18.80
**Canidae**	***Vulpes vulpes***	138.81	93.64	105.45	18.56	17.70
**Canidae**	***Canis lupus***	245.08	161.10	179.81	30.04	25.57
**Ursidae**	***Ursus arctos***	316.11	213.70	220.69	33.21	32.71

UDA—upper dental arch, and LDA—lower dental arch.

**Table 3:** Average values of bite force and bite force quotient on the canine and carnassial for Canidae, Felidae, and Ursidae representatives.

Taxa	CBF (N)	MBF (N)	CBFQ	MBFQ
***Panthera leo***	2608.77	4566.87	119.46	117.25
***Felis catus***	184.06	332.79	79.19	69.69
***Panthera tigris tigris***	3375.27	4172.26	193.16	132.59
***Canis lupus familiaris***	540.56	1100.59	90.42	94.17
***Vulpes vulpes***	344.35	774.15	141.29	155.17
***Canis lupus***	1141.26	2425.80	100.61	115.38
***Ursus arctos***	1627.01	3175.99	76.07	83.56

CBF—canine bite force; MBF—molar bite force; CBFQ—canine bite force quotient; MBFQ—molar bite force quotient.

**Table 4:** The average bending strength is in the AP (anteroposterior) and LM (lateromedial directions.

Taxa	SAP	SLM
*Panthera leo*	742.835	1781.659
*Felis catus*	1.077	1.706
*Panthera tigris tigris*	480.327	1273.253
*Canis lupus familiaris*	12.273	55.262
*Vulpes vulpes*	3.161	9.424
*Canis lupus*	86.194	316.215
*Ursus arctos*	740.227	2372.689

SAP—anteroposterior bending strength, and SLM—lateromedial bending strength.

## Data Availability

Access to the data will be granted upon request.
